# Research on Predicting the Occurrence of Hepatocellular Carcinoma Based on Notch Signal-Related Genes Using Machine Learning Algorithms

**DOI:** 10.5152/tjg.2023.22357

**Published:** 2023-07-01

**Authors:** Dingzhong Zhou, Sujuan Cao, Hui Xie

**Affiliations:** 1 Department of Interventional Vascular Surgery, Affiliated Hospital (Clinical College) of Xiangnan University, Chenzhou, P. R. China; 2 Key Laboratory of Medical Imaging and Artifical Intelligence of Hunan Province, Chenzhou, P. R. China; 3 Department of Oncology, Affiliated Hospital (Clinical College) of Xiangnan University, Chenzhou, P. R. China; 4 Department of Radiation Oncology, Affiliated Hospital (Clinical College) of Xiangnan University, Chenzhou, P. R. China

**Keywords:** Classification and diagnosis model, hepatocellular carcinoma, machine learning algorithms, notch signal-related genes

## Abstract

**Background/Aims:**

Hepatocellular carcinoma, a highly malignant tumor, is difficult to diagnose, treat, and predict the prognosis. Notch signaling pathway can affect hepatocellular carcinoma. We aimed to predict the occurrence of hepatocellular carcinoma based on Notch signal-related genes using machine learning algorithms.

**Materials and Methods:**

We downloaded hepatocellular carcinoma data from the Cancer Genome Atlas and Gene Expression Omnibus databases and used machine learning methods to screen the hub Notch signal-related genes. Machine learning classification was used to construct a prediction model for the classification and diagnosis of hepatocellular carcinoma cancer. Bioinformatics methods were applied to explore the expression of these hub genes in the hepatocellular carcinoma tumor immune microenvironment.

**Results:**

We identified 4 hub genes, namely, LAMA4, POLA2, RAD51, and TYMS, which were used as the final variables, and found that AdaBoostClassifie was the best algorithm for the classification and diagnosis model of hepatocellular carcinoma. The area under curve, accuracy, sensitivity, specificity, positive predictive value, negative predictive value, and F1 score of this model in the training set were 0.976, 0.881, 0.877, 0.977, 0.996, 0.500, and 0.932; respectively. The area under curves were 0.934, 0.863, 0.881, 0.886, 0.981, 0.489, and 0.926. The area under curve in the external validation set was 0.934. Immune cell infiltration was related to the expression of 4 hub genes. Patients in the low-risk group of hepatocellular carcinoma were more likely to have an immune escape.

**Conclusion:**

TheNotch signaling pathway was closely related to the occurrence and development of hepatocellular carcinoma. The hepatocellular carcinoma classification and diagnosis model established based on this had a high degree of reliability and stability.

**Main Points**

- We aimed to predict the occurrence of hepatocellular carcinoma (HCC) based on Notch signal-related genes using machine learning algorithms.
- We identified 4 hub genes, namely, LAMA4, POLA2, RAD51, and TYMS, which were used as the final variables, and found that AdaBoostClassifie was the best algorithm for the classification and diagnosis model of HCC.
- The Notch signaling pathway was closely related to the occurrence and development of HCC. The HCC classification and diagnosis model established based on this had a high degree of reliability and stability.

## Introduction

Primary liver cancer is one of the most common malignant tumors that seriously threaten human health in the world, and hepatocellular carcinoma (HCC) is the most common type of primary liver cancer. The latest report^1^ released by the International Anti-Cancer Alliance shows that primary liver cancer is the fifth most likely malignant tumor occurring in men and the seventh most likely malignant tumor occurring in women. Currently, the global incidence of HCC is increasing year by year, and there are 1 million new cases of HCC each year.^2^ The main treatment for HCC is surgical resection. Although surgical treatment may be effective for early HCC, the overall 5-year survival rate of patients is only 50%-70%.^3^ Moreover, up to 60%-70% of patients experience tumor recurrence within 5 years after surgery, and the long-term prognosis after hepatectomy is poor.^4^ Therefore, early diagnosis and early treatment of HCC are particularly important for the prognosis of HCC, and it is necessary to develop a new model to predict the occurrence of HCC.

The Notch signaling pathway is a classic signaling pathway, and its family members are highly conserved in structure. With the in-depth study of the Notch signaling pathway, it is found that this pathway plays an important role in the occurrence and development of tumors.^5^ It has been reported that the Notch signaling pathway can promote the development of cervical cancer cells, resulting in the formation of tumors.^5^ Similarly, downregulation of Notch1 expression in pancreatic cancer can significantly inhibit the growth of pancreatic cancer cells, promote cell apoptosis, and stop the cell cycle at G0-G1.^6^ Gramantieri et al^7^ found that the expression of Notch3 and Notch4 in liver cancer was significantly higher than that in adjacent tissues, Notch3 and Notch4 were also expressed in normal liver tissues and chronic hepatitis tissues, and that the Notch signaling pathway may participate in the invasion and metastasis of liver cancer. However, it is unclear whether Notch signal-related genes (NSRGs) are related to the prognosis of HCC, and it is necessary to further explore the exact relationship between immune infiltrating cells in the HCC microenvironment and NSRGs. With the rapid development of machine learning algorithms, we used it to study the above 2 problems and write this paper.

## Materials and Methods

### Data Collection

In October 2021, we downloaded the data of 424 HCC cases (tumor tissue: 374 cases, normal tissues: 50 cases) from The Cancer Genome Atlas (TCGA) database (https://tcga-data.nci.nih.gov/tcga/) as a training set and main research cohort. Then, we downloaded the HCC patient data (normal tissues: 192, tumor tissues: 240) of the GSE36376 and platform GPL10558433 from the Gene Expression Omnibus (GEO) database (https://www.ncbi.nlm.nih.gov/geo/) as an external validation data set.

We downloaded 237 NSRGs from the Molecular Signatures Database (MSigDB) (http://www.gsea-msigdb.org/gsea/msigdb) for research.

We used the R language to extract the expression matrix of NSRGs. All expression data were standardized by the Z-score processing (the mean value of the sample becomes 0, and the variance becomes 1). Notch signal-related genes were set as independent variables (feature) and normal samples/tumor samples as dependent variables (label) for the occurrence of HCC.

### Differential Expression Analysis of Notch Signal-Related Genes

We used the “limma” package of R language to select the differentially expressed NSRGs (DENSRGs) of HCC, with the criteria of |logFC| > 1 and FDR < 0.05 (FC is fold change and FDR is false discovery rate).

### Identification of Hub Notch Signal-Related Genes

In the TCGA-HCC cohort, 2 machine learning algorithms of least absolute shrinkage and selection operator (LASSO) and Support Vector Machine Recursive Feature Elimination (SVM-RFE) were used to screen the important NSRGs of HCC.

Learning algorithms of least absolute shrinkage and selection operator is a kind of regression analysis algorithm that selects variables while regularizing. It was implemented by the “glmnet” package of R language with parameter settings of family:binomial, alpha:1, type, measure:deviance, nfolds:10.

SVM-RFE can express complex classification boundaries by combining with the kernel function. It was implemented by the “e1071, kernalb, caret” packages of R language with parameter settings of functions: careFuncs, method: cv, methods: svmRadial. In order to avoid the over-fitting of the model, we also performed a univariate regression analysis of the gene in the selection of the feature genes using the “survival, survminer” packages of R language with the filter condition of *P* < .05.

The intersecting genes of the 3 methods were identified as the final feature genes for further research as the variables of the classification and diagnosis model.

### Establishment and Verification of the Prognostic Model

The model of HCC classification and diagnosis was constructed based on the expression of core genes. In this study, 5 classification algorithms of machine learning classification algorithms, including XGBClassifier, LGBMClassifier, AdaBoostClassifier, MLPClassifier, and SVM, were used to construct the initial classification and diagnosis model.

About 30% of TCGA-HCC patients were randomly selected as the test set and the remaining 70% as the training set for the 10-fold cross-validation, and GEO-HCC patients as the external validation set were used to validate the model. Model evaluation indicators included area under curve (AUC), accuracy, sensitivity, positive predictive value (PPV), negative predictive value (NPV), and F1 score. The number of true positive, true negative, false positive, and false negative samples were represented as TP, TN, FP, and FN, respectively. A comprehensive evaluation of various indicators and selection of the best algorithm were done to build the model.

Accuracy = (TP + TN)/(TP + TN + FP + FN)

Sensitivity = TP/(TP + FN)

F1 = 2TP/(2TP + FN + FP)

PPV = TP/(TP + FP)

NPV = TN/(TN + FN)

XGBClassifier was implemented based on the “xgboost 1.2.1” package of python language, and this model parameters were objective (optimized objective function): binary, logistic, learning_rate (learning rate): 0.3, max_depth (maximum tree depth): 4, min_child_weight (minimum bifurcation weight sum): 6, and reg_lambda (L2 regularization coefficient): 1. The LGBMClassifier machine learning model was based on the “lightgbm 3.2.1” package of python language, and this model parameters were boosting_type (algorithm type): gbdt, learning_rate (learning rate): 0.001, max_depth (maximum tree depth): 1, n_estimators (maximum number of trees): 5, and num_leaves (the maximum number of leaves): 5. AdaBoostClassifier was implemented based on the “sklearn 0.22.1” package of python language, and this model parameters were learning_rate (learning rate): 0.1, and n_estimators (number of single models): 50. MLPClassifier was implemented based on the “sklearn 0.22.1” package of python language, and this model parameters were activation (non-linear function): logistic, hidden_layer_sizes (hidden layer width): (30, 30), and max_iter (number of iterations): 10. Support vector machine was implemented based on the “sklearn 0.22.1” package of python language, and this model parameters were C (regularization factor): 0.1, kernel (core type): rbf, tol (convergence measure): 0.001.

### Hub Gene Expression Analysis

In order to further analyze the relationship between participating variables (hub genes) and HCC. The Mann–Whitney *U-*test was used to compare the expression levels of hub genes between tumor group and normal group. Pearson’s correlation was used to calculated the correlation of risk genes.

### Immune Cell Infiltration Analysis

The CIBERSORT algorithm was used to evaluate the infiltration of 22 immune cells in the TCGA-HCC cohort. Then, we compared the distribution of the 22 immune cells between normal group and tumor group. Spearman’s correlation analysis was performed between 22 immune cells and hub genes. In the end, the risk of patients was scored according to the gene expression of the selected variables in the model. The patients were divided into the high-risk group and the low-risk group by the median value of the risk score and then analyzed for immunotherapy responsiveness. The risk score was calculated as the sum of the predicted values weighted by the LASSO coefficient, including all risk genes. The Tumor Immune Dysfunction and Exclusion (TIDE) tool (http://tide.dfci.harvard.edu/) was used to predict immunotherapy responsiveness.

## Results

**Figure 1.**
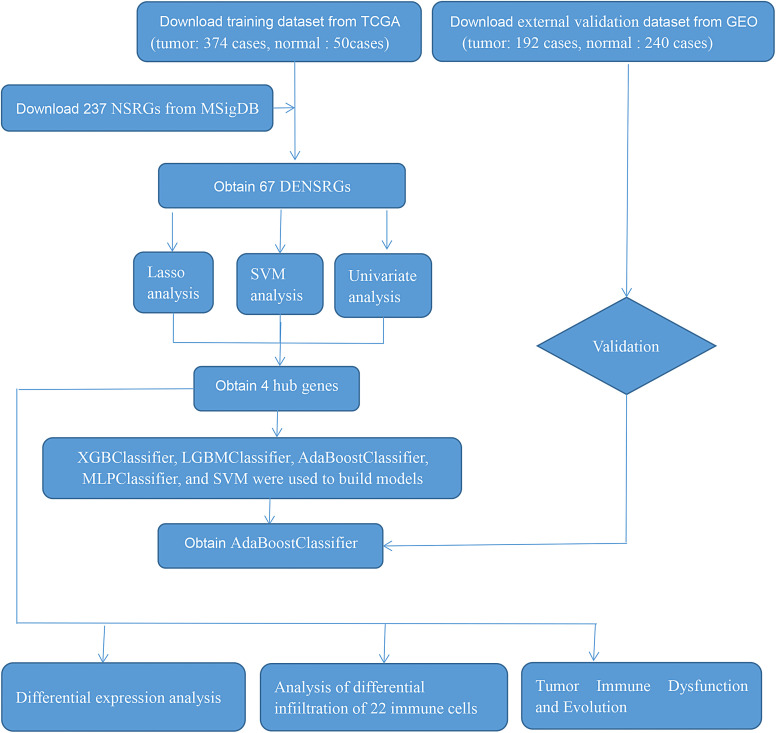
The flowchart of the whole study.

The flowchart of this study is shown in Figure 1.

### Differential Expression Genes Analysis

Sixty-seven DENSRGs were identified with |logFC| > 1 and FDR < 0.05; of which, 4 genes were downregulated and 63 genes were upregulated (Supplementary Material 1).

### Identification of Hub Genes

**Figure 2.**
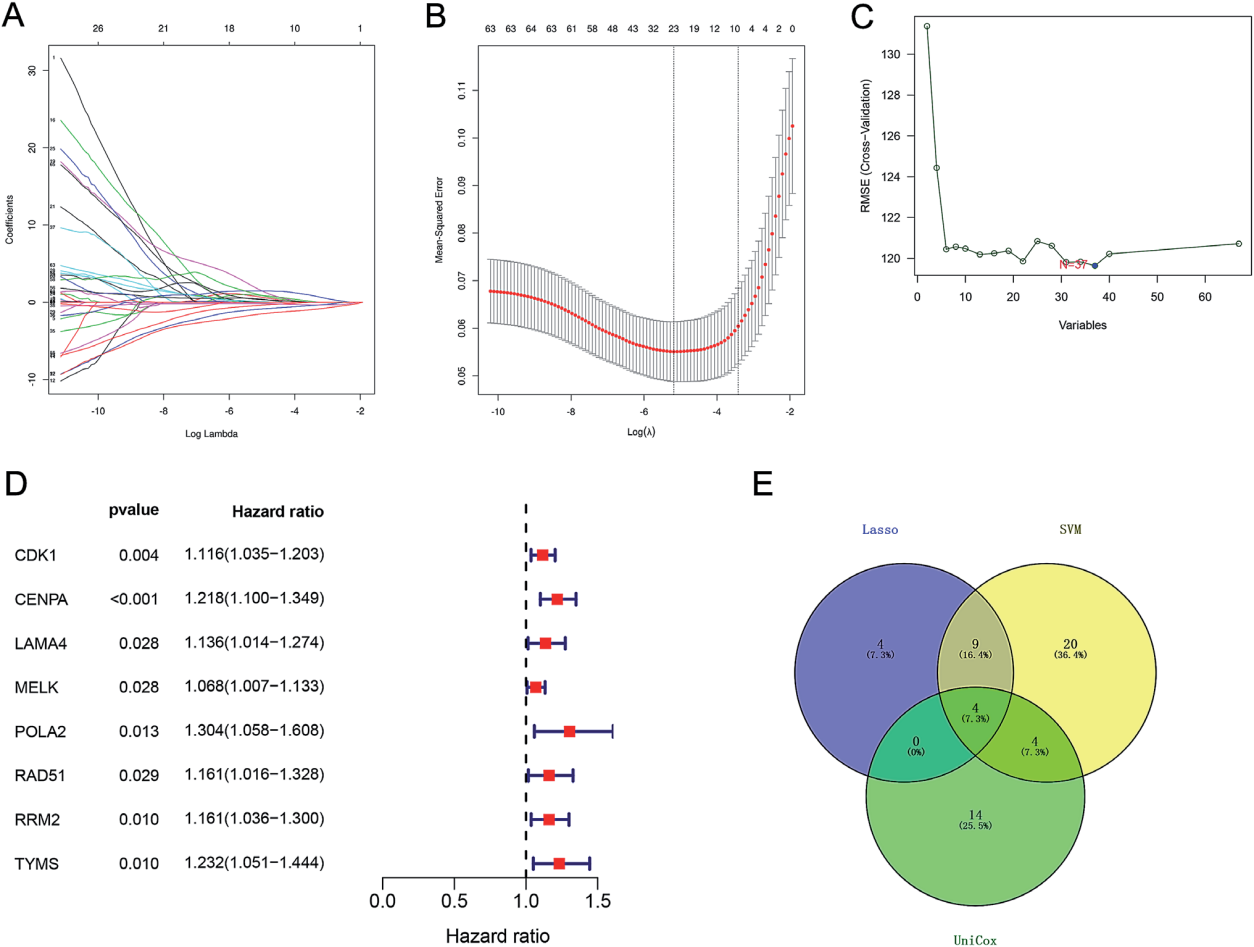
Variables screening process. (A) Trend graph of LASSO coefficients; (B) partial likelihood deviation map; (C) using SVM-RFE feature selection; (D) univariate cox regression analysis; (E) Venn diagram of overlapping genes. SVM, support vector machine; LASSO, least absolute shrinkage and selection operator.

The LASSO algorithm analyzed the DENSRGs of the TCGA-HCC cohort to select key feature genes and determine the optimal value λ with the smallest mean square error through 10-fold cross-validation (Figure 2A). When λ was 0.006, 17 feature genes were screened out (Figure 2B). The main use of the SVM algorithm is for the 2 classification problems, find a hyperplane and divide the 2 categories to ensure the minimum classification error rate. SVM-REF analysis showed that 37 genes were closely related to HCC (Figure 2C). Hepatocellular carcinoma univariate regression analysis found that there were 18 NSRGs related to the survival of HCC (Figure 2D). The intersection of the 3 methods finally resulted in 4 hub genes: LAMA4, POLA2, RAD51, and TYMS (Figure 2E).

**Figure 3.**
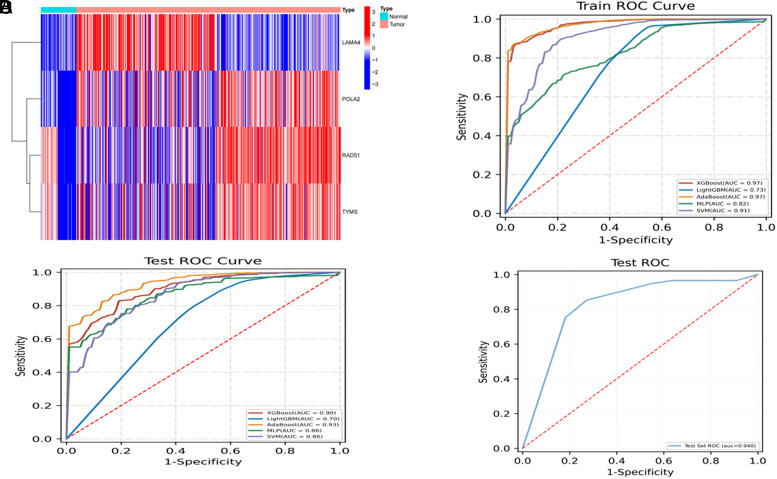
Establishment of model. (A) Heat map of model variables; (B) ROC curves of classification model of HCC in training set; (B) ROC curves of classification model of HCC in test set; (D) ROC curves of AdaBoostClassifier model of HCC in external data test set. HCC, hepatocellular carcinoma; ROC, receiver operating curve.

In our present study, the classification and diagnosis model of HCC was constructed based on the expression levels of LAMA4, POLA2, RAD51, and TYMS (Figure 3A). We chose the best among the 5 machine learning classification algorithms of XGBClassifier, LGBMClassifier, AdaBoostClassifier, MLPClassifier, and SVM by the metrics of AUC, accuracy, sensitivity, PPV, NPV, and F1 score. The best performer in the training set was AdaBoostClassifier (Figure 3B), and the corresponding scores in the training set in each evaluation standard were AUC: 0.976, accuracy: 0.881, sensitivity: 0.877, specificity: 0.977, PPV: 0.996, NPV: 0.500, and F1 score: 0.932 (Table 1). The best performer in the testing set was also AdaBoostClassifier (Figure 3C), and the corresponding scores in the testing set in each evaluation standard were AUC: 0.934, accuracy: 0.863, sensitivity: 0.881, specificity: 0.886, PPV: 0.981, NPV: 0.489, and F1 score: 0.926 (Table 2). The results in the training set were consistent with those in the testing set, and AdaBoostClassifier was considered as the best model.

### Validation model

We used GEO-HCC cohort data to verify the classification and diagnosis model constructed by the AdaBoostClassifier method. All patients were verified as AUC = 0.940 in the HCC tumor and normal tissue classification model (Figure 3D).

### Gene Expression Analysis

**Figure 4.**
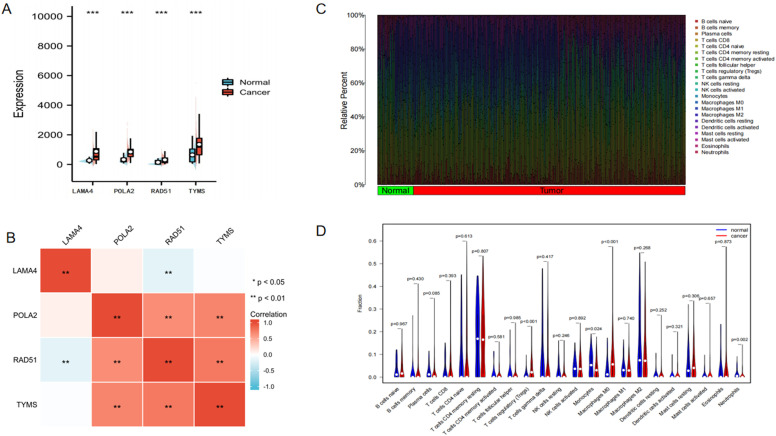
Bioinformatics analysis, (A) the expression of 4 genes difference between normal and tumor tissues; (B) graphs depicting significant associations between 4 genes; (C) bar charts of 22 immune cell proportions in HCC and normal tissues; (D) difference analysis of 22 immune cells infiltration between HCC and normal tissues. HCC, hepatocellular carcinoma.

The 4 risk genes of LAMA4, POLA2, RAD51, and TYMS showed significant differences in the expression of normal and tumor tissues, and the 4 genes showed high expression in tumor tissues (Figure 4A). We found that POLA2 and TYMS were highly positively correlated with RAD51, while LAM4 was highly negatively correlated with RAD51(Figure 4 B).

**Figure 5.**
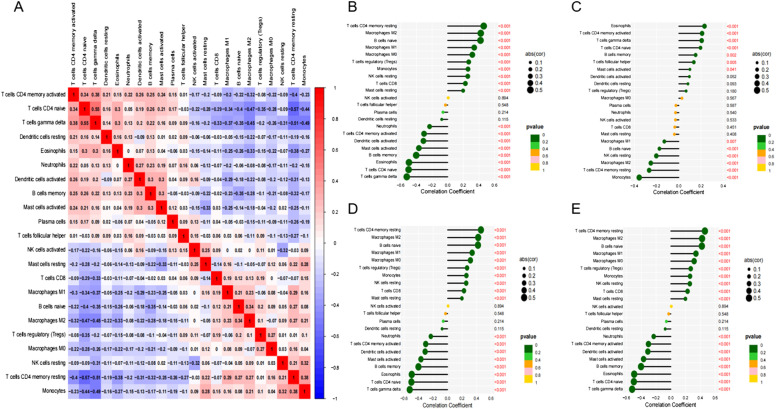
(A) Graphs depicting significant associations between 22 immune cells infiltration; (B) correlation between LAMA4 and immune cells infiltration; (C) correlation between POLA2 and immune cells infiltration; (D) correlation between RAD51 and immune cells infiltration; (E) correlation between TYMS and immune cells infiltration.

**Figure 6.**
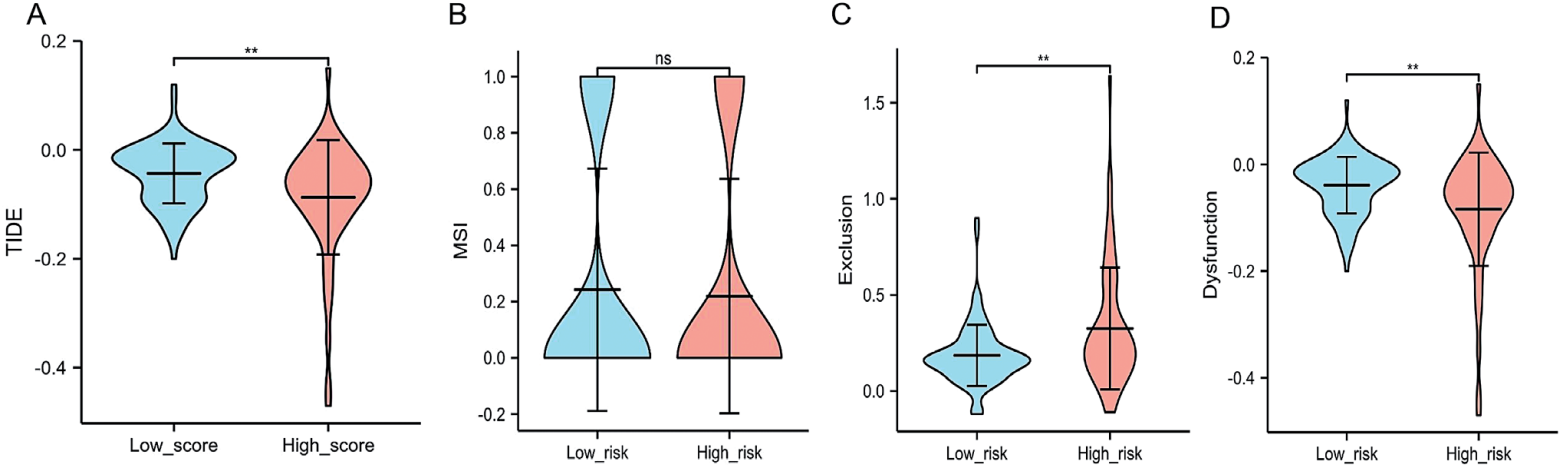
(A) Difference analysis of TIDE between low and high risk of HCC; (B) difference analysis of MSI between low and high risk of HCC; (C) difference analysis of exclusion between low and high risk of HCC; (D) difference analysis of dysfunction between low and high risk of HCC. TIDE, Tumor Immune Dysfunction and Exclusion; HCC, hepatocellular carcinoma.

In the analysis of the difference in immune cell infiltration between normal and tumor tissues, Tregs (*P* < .001), monocytes (*P* = .024), macrophages (*P* < .001), and neutrophils (*P* = .002) showed differences (Figure 4C and 4D). The correlation analysis between immune infiltrating cells suggested that macrophages and Tregs were negatively correlated with T cells (Figure 5A). The correlation analysis between risk genes and immune cell infiltration subtypes found that NK cells activated had no correlation with LAMA4, POLA2, RAD51, and TYMS. However, almost all of the other immune cell infiltration subtypes showed varying degrees of correlation with risk genes (Figure 5B-5E). We used the public website http://tide.dfci.harvard.edu to perform an analysis of TIDE and microsatellite instability (MSI) immunotherapy of HCC and found that HCC patients showed differences in TIDE between the high-risk group and the low-risk group (Figure 6A-6D).

## Discussion

The mortality of HCC ranks second among all kinds of cancers, and the new cases of HCC in China each year accounts for more than half of new cases around the world.^8^ The treatment of HCC is affected by liver function, nodule size, metastasis, and age. The Notch signaling pathway as a classic signaling pathway can regulate the occurrence of tumor cells.^9^ There is evidence that the Notch signaling pathway is extraordinarily active in HCC.^10^ At present, surgical resection is still the main option for HCC, but its recurrence risk is very high. With the help of machine learning methods, the prediction accuracy of early HCC can be improved, thereby further improving the treatment outcome of HCC patients.

In our present study, we firstly used LASSO and SVM-REF algorithms combined with univariate survival analysis to study the differential expression matrix of NSRGs in the TCGA-HCC cohort. Then, after comparing the results of 5 machine learning classification algorithms, we finally decided to use AdaBoostClassifier to establish the HCC classification and diagnosis model. The HCC classification and diagnosis model established in our present study had an AUC of 0.976 ± 0.007 in the training set and 0.934 ± 0.033 in the test set. In the external data test of the GEO-HCC cohort, this model had an AUC of 0.934, an accuracy of 0.863, the sensitivity of 0.881, specificity of 0.886, PRV of 0.981, NPV of 0.489, and F1 score of 0.926. These results showed that the classification and diagnosis model established in our present study might be highly reliable. Duan et al^11^ used the biological processes (BP) neural network to establish an auxiliary diagnosis model of lung cancer with genes of P16, RASSF1A, and FHIT, and the AUC of this model was 0.76. Sherafatian and Arjand et al^12^ used the decision tree method and lung adenocarcinoma and lung squamous cell carcinoma data sets from TCGA to construct a diagnostic model for classifying the sample as lung squamous cell carcinoma, and the AUC of this model was 0.916. Yu et al^13^ used computed tomography imaging data combined with machine learning methods to diagnose lung cancer patients and determine their pathological stages, and the AUC of the final model ranged from 0.69 to 1.00. It can be seen that the machine learning method can solve the classification problem of lung cancer very well. In our present study, the HCC diagnosis model established by AdaBoostClassifier had an AUC of 0.976 ± 0.007 in the training set, 0.934 ± 0.033 in the testing set, and 0.932 in the external validation set, which suggested that our presented study has certain advantages and reliability compared with similar models.

Studies have shown that the downregulation of LAMA4 expression can inhibit the proliferation and migration of breast cancer, renal cell carcinoma, gastric cancer, and ovarian cancer.^14-16^ Our study showed that LAMA4 was highly expressed in HCC tumor tissues. Considering that LAMA4 is closely related to the migration of cancer cells and tumor progression in a series of tumors, the latest research describes LAMA4 as “oncolaminin.”^17^ The crosstalk between Notch and TGF-β1 has been reported many times. It has been reported that LAMA4 could affect the level of Notch ligand and its receptor by regulating TGF-β1,^18^ thereby inducing the expression of some key proteins related to the occurrence and development of HCC. Cir_POLA2 has been reported as an oncogene of lung cancer.^19^ It has been found that overexpression of Cir_POLA2 can promote the proliferation of acute myeloid leukemia cells.^20^ Circ_POLA2 may upregulate the G protein subunit beta 1 (Notch pathway-related molecules) by serving as an endogenous competing RNA for miR-326.14.^21^ Guanine nucleotide regulatory protein (G protein) is the core of normal liver cell function and is related to the occurrence and progression of liver disease. It was reported that the G protein family was involved in the development of HCC.^22^ In our present study, when we performed the comparison between liver tumor tissue and paracancerous tissue, it was found POLA2 was relatively lower in the normal paracancerous tissues. As we know, the DNA repair protein RAD51 mainly plays an important role in maintaining genome stability and regulating the cell life cycle. It has been reported that the DNA repair system in most HCC cells is extraordinarily active, resulting in poor therapeutic effect of HCC.^23^ RAD51 is a key protein for DNA double-strand repair. Highly expressed RAD51 promotes the repair of HCC.^24^ Chen et al^25^ have reported that in female ovarian cancer, knocking down the expression of RAD51 can significantly reduce the proliferation rate of ovarian cancer cells. It has been found that the high expression of RAD51 is related to the higher pathological grade and clinical stage of HCC, and it is an independent risk factor affecting the overall survival and prognosis of HCC.^26^ TYMS is the key rate-limiting enzyme that controls the synthesis of dTMP. The synthesis of dTMP is closely related to functions such as DNA synthesis, replication, and repair. Therefore, TYMS is currently considered to be the next anti-tumor target that is most likely to be successfully developed.^27^ Studies have shown that the activity of thymidylate synthase in many patients with malignant tumors is significantly higher than that in normal tissues.^27^ TYMS can regulate the growth of tumor cells by affecting the expression and expression cycle of P53, so TYMS is related to the proliferation status of malignant tumors.^28^ Studies have shown that in most of the tumor cells with growth advantages, TYMS is overexpressed, and the higher the expression of TYMS is, the worse the prognosis of patients is.^28^ A study showed that the positive expression rate of TYMS in liver cancer tissues was significantly higher than that in the control group and adjacent tissues, and the high expression of TYMS indicated that the tumor was more aggressive.^29^

Immune infiltrating cells are an important part of the tumor microenvironment (TME) which are closely related to the progression of HCC.^30^ In this study, we used the CIBERSORT algorithm to evaluate patients’ immune cell infiltration. Further analysis found that there were differences in T cells regulatory (Tregs), monocytes, macrophages M0, and neutrophils between normal tissues and tumor tissues (*P*  < .05). The proportion of Tregs and macrophages M0 in tumor tissues is higher than that in normal tissues, and Tregs are strongly positively correlated with macrophages M0, while the proportion of monocytes and neutrophils in tumor tissues is lower than that in normal tissues, and monocytes are strongly negatively correlated with neutrophils. The increase of macrophages M0 is significantly correlated with OS and tumor stage of HCC.^31^ Macrophages M0 can stimulate the production of TAM and Kupffer cells in the presence of carcinogenic factors, thereby inhibiting the progression of HCC caused by immunity.^32^ This may be related to the malignant behavior of the highly expressed genes from our research and analysis. The correlation analysis of our present study also confirmed the significant positive correlation between LAMA4, RAD51, and TYMS and macrophages M0. It has been reported that TGF-β1 is strongly positively correlated with macrophage in HCC.^33^ Macrophages M0 can secrete a large amount of TGF-β1.^33^ In our study, it was found that LAMA4 affected the progress of HCC by regulating TGF-β1. This also shows the correctness of this research. Under normal circumstances, Tregs inhibit the anti-autoimmune response and play an important role in balancing immune tolerance and inflammation. It has been reported that the upregulation of Tregs is a predictor of adverse outcomes in HCC patients.^34^ It has been reported that Tregs promote the migration and invasion of liver cancer cells through epithelial-to-mesenchymal transition induced by TGF-β1.^35^ Neutrophils are the most common white blood cells in circulation. They play an important role in host defense, immune regulation, and tissue damage. Neutrophils are considered to be one of the first immune cells that enter the TME and interact with cancer cells. Thus, it plays an important role in the progression of cancer. Our research findings are similar to other studies. The content of neutrophils in normal tissues is high, and it is significantly negatively correlated with LAMP4, RAD51, and TYMS. In our present study, we found that RAD51 was strongly positively correlated with TYMS, and this interrelationship further strengthens the immune function of neutrophils.

The liver has a special population of immunosuppressive cells, which can avoid liver damage caused by autoimmunity and chronic inflammation under normal physiological conditions. But for patients with liver cancer, these special cells can cause tumor immune escape and promote disease progression. Our present study found that TIDE in the low-risk population of HCC was all higher than those in the high-risk population of HCC (*P*  < .05). This showed that immune escape was prone to occur in the low-risk population. The previous analysis showed that Tregs cells are abundant in HCC tumors and are a subset of CD4+ T cells, a type of lymphocyte with high immunosuppressive properties.^36^ They suppress the immune response by inhibiting CD8+ T cell effector functions and directly promote tumor escape through a variety of contact-dependent and non-contact mechanisms.^37^ In HCC, neutrophils can recruit macrophages and Tregs into HCC by releasing cytokines, thereby promoting tumor progression and developing resistance to sorafenib.^38^

There are some limitation to our study. The biological functions of LAMA4, POLA2, RAD51, and TYMS need to be further explored by experiments. The construction and validation of our established model are only based on the public databases, and thus it is necessary to use more clinical research data to further validate clinical efficacy of this model.

In conclusion, the classification and diagnosis model of HCC based on NSRGs in our presente study showed that the Notch signal was related to the occurrence and development of HCC. The classification diagnosis model might effectively distinguish HCCs from healthy patients. This study also suggested the role of immune infiltrating cells in the occurrence and development of HCC. Our findings provide a new method for the diagnosis and prognosis of HCC from the perspective of immune cell infiltration.

## Figures and Tables

**Supplementary Material 1.** Specific Information About 67 Differentially Expressed Notch Signal-Related Genes

Gene	Group1mea	Group2mea	LogFC	*P*	FDR
ADCK2	317.94	777.438502673797	1.28997402576259	.00662769612589925	0.0226528718332974
ALB	3249800.64	1 311 646.06951872	−1.30897273815077	.0110076236156233	0.0336099441063698
ALPP	0.06	1.04812834224599	4.12670907328114	.00601992768724134	0.0208873248542162
AP1S1	594.82	1388.36631016043	1.22286320058912	.0016648296735327	0.00706011102294423
BIRC5	23.9	774.360962566845	5.0179237013357	.000101553631795433	0.00122398850953443
BUB1	11.44	251.013368983957	4.4556052469998	.000965797747537303	0.00543153858520314
CAPG	229.56	916.310160427807	1.99696473711372	.019419003938911	0.0499657517079845
CCNA2	18.08	445.29679144385	4.62230063371022	.00123412247815994	0.00585089535491346
CCNB2	13.48	321.673796791444	4.57670601872659	5.75072935886893e-08	1.64614627897623e-06
CDC20	16.92	618.55614973262	5.19210308891322	.000822342319485665	0.00523101086561715
CDC25C	3.58	139.893048128342	5.28822087300828	.00019366849370602	0.00187257345333701
CDC45	10.18	189.799465240642	4.22066646105005	.00112210110525322	0.005474235487487
CDC6	19.18	395.906417112299	4.36748482624149	.000995538485106148	0.00543153858520314
CDCA8	16.88	273.283422459893	4.01701113859114	5.90968954848756e-05	0.000796069945060971
CDK1	120.36	512.631016042781	2.09056475778866	8.52692437483536e-05	0.00108481426768739
CDKN3	9.64	264.358288770053	4.77731760544587	.000585110254082583	0.00463071816074427
CENPA	14.62	121.171122994652	3.05103070620143	4.93753583158657e-16	5.65347852716662e-14
CENPM	10.34	208.045454545455	4.33059067742315	.000645387776444793	0.00463071816074427
COCH	5.22	158.467914438503	4.92399714532306	6.15410883818011e-09	2.34881820657208e-07
DLX4	7.24	25.7967914438503	1.83313003470397	.00214441296258008	0.00892855578965161
E2F1	22.8	656.820855614973	4.84839420731861	.0013797908738887	0.00616161045625581
E2F2	5.3	82.0561497326203	3.95254719638843	.000951800369312027	0.00543153858520314
E2F8	4.56	100.470588235294	4.46159559398013	.000357593832420774	0.00303292546756879
FCAR	7.5	2.61497326203209	−1.52009440054392	.0161711327742108	0.0451608464060277
FEN1	158.5	903.251336898396	2.5106446446213	.000937078990907774	0.00543153858520314
GDF3	17.9	50.9171122994652	1.5081910134195	.0142555177988104	0.0413229566573112
GINS2	43.62	252.532085561497	2.53340502659355	.00348106611789121	0.0135112566270693
GM2A	861.34	1900.6871657754	1.1418663626169	.00694497244882323	0.02338821604089
GTSE1	7.42	163.641711229947	4.46297753186007	.000689460178201999	0.00478443578206842
HMMR	16.2	259.489304812834	4.00160935904477	.000632502493502348	0.00463071816074427
IFNA4	24.28	3.33689839572193	−2.86318875750635	.000291892515067697	0.0026737354380201
KIF20A	12.56	328.377005347594	4.70844473635916	.000996177382439003	0.00543153858520314
KIFC1	17.62	459.77807486631	4.7056518414789	.000647087253903129	0.00463071816074427
LAMA4	268.82	877.03743315508	1.70599794267374	4.62249090179729e-11	3.52850138837193e-09
LGALS2	126.4	318.657754010695	1.33401130326571	.000316257794889145	0.00278550134729285
LIG1	239.2	863.352941176471	1.85173306835301	.00267426221147406	0.0107439657267993
LMNB1	169.58	720.377005347594	2.08678609997913	.00508250325675122	0.0184744959650163
MELK	10.3	227.96256684492	4.46808070002267	1.15535165862604e-09	6.61438824563408e-08
MMP9	59.14	423.104278074866	2.83880712451689	.00342341503871463	0.0135112566270693
NAP1L1	2000.54	4888.2486631016	1.28892820121369	.0176640083201824	0.0481554512538306
NCAPG	11.52	302.25935828877	4.71357438572973	3.87672104072929e-07	8.87769118327007e-06
NIPAL3	122.1	288.072192513369	1.23836720415278	.00110235574258372	0.005474235487487
ORC1	8.2	128.756684491979	3.97287961279108	.00109361566036424	0.005474235487487
PALB2	112.18	245.743315508021	1.13133668605473	.00711120751110934	0.0236009640586093
PASK	51.92	160.927807486631	1.63205134933818	.0161211279578174	0.0451608464060277
PCLAF	27.96	269.422459893048	3.26843385752688	.00125193830738323	0.00585089535491346
PLK1	17.18	385.946524064171	4.48959902297166	.00110624468794798	0.005474235487487
POLA2	325.36	845.94385026738	1.3785250113697	7.18250805478335e-18	1.64479434454539e-15
POLM	1717.6	3581.75401069519	1.06027216236116	1.95450301558996e-05	0.000298387460380067
PRUNE1	299.66	805.355614973262	1.42629944507522	.00019625223965104	0.00187257345333701
RAD51	141.44	310.935828877005	1.13642668696551	1.93123975677148e-08	6.31791291858098e-07
RRM2	570.42	1175.74598930481	1.04347993950693	1.28405633607606e-05	0.000222786007811987
S100A12	17.76	3.50534759358289	−2.34100216624126	.00439438496856373	0.0162308735129209
SAC3D1	117.18	452.767379679144	1.9500436656767	.00112353304765017	0.005474235487487
SHCBP1	6.2	136.430481283422	4.4597539814203	.000992426546098801	0.00543153858520314
SLC22A4	11.94	80.475935828877	2.75275461183624	.0128337352148675	0.0381678618727878
SMC2	120.78	381.358288770053	1.65876547878417	.00928904053291306	0.0291395929046177
SPATA6	65.9	172.27807486631	1.38638873693743	.000153475772365575	0.00175729759358583
THOC5	226.1	652.930481283422	1.52996840144838	.00139682544522702	0.00616161045625581
TMEM161A	657.7	1410.74064171123	1.10095120396756	.0108594361179344	0.0336055523109051
TOP2A	50.26	1308.48663101604	4.70234470280807	.000589599494019082	0.00463071816074427
TYMS	641.98	1352.57754010695	1.07511104476597	5.16881322531014e-09	2.34881820657208e-07
USP46	99.34	210.016042780749	1.08005288531888	.0193447957975716	0.0499657517079845
VAMP1	50.12	160.954545454545	1.68319500020648	.00242449547379185	0.00991445470532739
VDR	50.22	176.197860962567	1.81086247636245	2.65270814711254e-05	0.000379668853555482
ZNF589	43.64	129.192513368984	1.56579946234137	.00139914298570001	0.00616161045625581
ZWINT	48.76	501.708556149733	3.36307951099703	.000813870556336174	0.00523101086561715

**Table 1.** Performance of the Classification and Diagnosis Models based on 5 Machine Learning Algorithms in the Training Set

Model	AUC	Accuracy	Sensitivity	Specificity	PPV	NPV	F1
XGBClassifier	0.973 ± 0.007	0.893 ± 0.030	0.890 ± 0.035	0.959 ± 0.048	0.994 ± 0.006	0.529 ± 0.094	0.939 ± 0.018
LGBMClassifier	0.731 ± 0.027	0.120 ± 0.007	0.957 ± 0.021	0.505 ± 0.073	N/A	0.120 ± 0.007	N/A
AdaBoostClassifier	0.976 ± 0.007	0.881 ± 0.020	0.877 ± 0.024	0.977 ±0.031	0.996 ± 0.005	0.500 ± 0.057	0.932 ± 0.013
MLPClassifier	0.822 ± 0.061	0.705 ± 0.090	0.691 ± 0.107	0.832 ± 0.058	0.968 ± 0.010	0.285 ± 0.077	0.802 ± 0.073
SVM	0.908 ± 0.016	0.873 ± 0.011	0.882 ± 0.012	0.840 ± 0.037	0.976 ± 0.006	0.480 ± 0.036	0.926 ± 0.007

HCC, hepatocellular carcinoma; AUC, area under curve; PPV, positive predictive value; NPV, negative predictive value; SVM, support vector machine; N/A, not available.

**Table 2.** Performance of the Classification and Diagnosis Models Based on 5 Machine Learning Algorithms in the Testing Set

Model	AUC	Accuracy	Sensitivity	Specificity	PPV	NPV	F1
XGBClassifier	0.901 ± 0.048	0.844 ± 0.065	0.853 ± 0.081	0.854 ± 0.105	0.975 ± 0.019	0.470 ± 0.114	0.907 ± 0.046
LGBMClassifier	0.699 ± 0.051	0.113 ± 0.017	0.946 ± 0.036	0.451 ± 0.124	N/A	0.113 ± 0.017	N/A
AdaBoostClassifier	0.934 ± 0.033	0.863 ± 0.059	0.881 ± 0.077	0.886 ± 0.090	0.981 ± 0.016	0.489 ± 0.128	0.926 ± 0.042
MLPClassifier	0.867 ± 0.085	0.788 ± 0.148	0.785 ± 0.179	0.865 ± 0.118	0.980 ± 0.015	0.429 ± 0.195	0.859 ± 0.120
SVM	0.866 ± 0.064	0.867 ± 0.062	0.886 ± 0.068	0.791 ± 0.118	0.970 ± 0.017	0.487 ± 0.127	0.925 ± 0.040

HCC, hepatocellular carcinoma; AUC, area under curve; PPV, positive predictive value; NPV, negative predictive value; SVM, support vector machine; N/A, not available.
